# Supplementary material for: A Critical View of the Application of the APEX Software (Aqueous Photochemistry of Environmentally-Occurring Xenobiotics) to Predict Photoreaction Kinetics in Surface Freshwaters
Source: Molecules. 2019 Dec 18;25(1):9. doi: 10.3390/molecules25010009 (PMC7017383; doi:10.3390/molecules25010009)
Supplement: Supplementary file 1 [file molecules-25-00009-s001.zip › 3.2.4_gcc-4.4.0.zip!/3.2.4_gcc-4.4.0/3.2.4_gcc-4.4.0/doc/gnuplot/postscript-terminal/ps_file.doc]

A short guide to PostScript files created by gnuplot's "postscript" terminal

by Dick Crawford, aka rccrawford@lanl.gov

Before we begin, let me say a few words about PostScript. It is highly ver-

satile, but with versatility comes complexity. Syntax is extremely important;

a misplaced command can do unexpected things. Thus the adventurous soul who

wishes to alter the PostScript but has not been initiated into the arcana of

PostScript would be well-advised to change only those items (like numerical

values, font names or heights, or the letters defining symbols) that are

fairly obvious as to their meaning.

PostScript is stack-oriented and works in Reverse Polish notation:

'a b add' takes 'a' and 'b' off of the stack and replaces them with 'a+b'.

The 'stroke' command actually draws lines on the page. It uses whatever line

width and style are currently defined. Thus if we specify a bunch of commands

like 'LT1 1 2 moveto 3 4 lineto LT3 stroke' [LT1 and LT3 are line type defi-

nitions, 'x y moveto' moves the (virtual) pen to (x,y) and 'x y lineto' draws

a line from wherever the pen was to (x,y)], the line connecting points (1,2)

and (3,4) will be in type LT3.

PostScript is case-sensitive.

The sample file below has been pruned of repeated commands, just to keep its

length down. Comment lines begin with '%'. Lines beginning with '%%' are

inserted for "encapsulated PostScript" -- these lines are read by applications

that import PostScript. Lines beginning with '%#' have been added to the

sample by me for explanatory purposes.

I have merged files created by the postscript terminal with no options, with

the "eps" option and with the "enhanced" option. The differences will be

clearly pointed out when appropriate.

Here we go.

%!PS-Adobe-2.0

%%Title: psgenh

%%Creator: gnuplot 3.5 (pre 3.6) patchlevel beta 338

%%CreationDate: Fri Jan 16 13:18:18 1998

%%DocumentFonts: (atend)

%%BoundingBox: 50 50 554 770

%%Orientation: Landscape

%# In 'eps' mode, the preceding two lines would look like:

%# %%BoundingBox: 50 50 410 302

%# %%Orientation: Portrait

%%Pages: (atend)

%%EndComments

%#

%# The 'dictionary' contains stuff defined by the user.

%#

/gnudict 120 dict def

gnudict begin

%#

%# The following switch toggles between color and monochromatic.

%#

/Color false def

%#

%# The following switch toggles between solid and dot-dash lines.

%#

/Solid false def

%#

%# The following parameter scales all linewidths in the plot.

%#

/gnulinewidth 5.000 def

/userlinewidth gnulinewidth def

%#

%# The following parameter specifies the vertical displacement of the labels

%# and titles (it is used in the 'show' commands defined below). It should

%# be about 1/3 of the font height.

%#

/vshift -46 def

%#

%# The following parameter scales the lengths of all dot-dash patterns.

%#

/dl {10 mul} def

%#

%# The following two parameters scale the horizontal and vertical sizes of

%# the symbols used by 'plot with points'.

%#

/hpt_ 31.5 def

/vpt_ 31.5 def

/hpt hpt_ def

/vpt vpt_ def

%#

%# The following four commands are aliases of the four 'pen movement'

%# commands. Use of these aliases significantly shortens the file. Note

%# that the first two are 'absolute' movements and the latter two are

%# 'relative'. M and R move to the specified position; L and V draw a

%# straight line (from the current position) to it.

%#

/M {moveto} bind def

/L {lineto} bind def

/R {rmoveto} bind def

/V {rlineto} bind def

%#

%# These are a couple more parameters used in plotting symbols. Why

%# they are here instead of up with 'hpt' and 'vpt' I know not.

%#

/vpt2 vpt 2 mul def

/hpt2 hpt 2 mul def

%#

%# The 'show' command writes out a character string. The following three

%# varieties do so as left-, right-, and center-justified. [Remember,

%# we're still in the dictionary--the font doesn't need to be specified

%# until we actually use one of these.]

%#

/Lshow { currentpoint stroke M

0 vshift R show } def

/Rshow { currentpoint stroke M

dup stringwidth pop neg vshift R show } def

/Cshow { currentpoint stroke M

dup stringwidth pop -2 div vshift R show } def

%#

%# The following commands define the various line types (normal, bold,

%# dashed, etc.) used by gnuplot.

%#

%# UP, DL, and UL are busywork commands used by others here

%#

/UP { dup vpt_ mul /vpt exch def hpt_ mul /hpt exch def

/hpt2 hpt 2 mul def /vpt2 vpt 2 mul def } def

/DL { Color {setrgbcolor Solid {pop []} if 0 setdash }

{pop pop pop Solid {pop []} if 0 setdash} ifelse } def

/BL { stroke gnulinewidth 2 mul setlinewidth } def	%# twice the linewidth

/AL { stroke gnulinewidth 2 div setlinewidth } def	%# half the linewidth

/UL { gnulinewidth mul /userlinewidth exch def } def

/PL { stroke userlinewidth setlinewidth } def	%# normal linewidth

/LTb { BL [] 0 0 0 DL } def

/LTa { AL [1 dl 2 dl] 0 setdash 0 0 0 setrgbcolor } def

/LT0 { PL [] 0 1 0 DL } def

/LT1 { PL [4 dl 2 dl] 0 0 1 DL } def

%#

%# ...and a bunch more.

%# In the LT's, the first command ('PL' for LT1) sets the linewidth,

%# the stuff in [...] defines the dot-dash pattern, and the three numbers

%# define the rgb color.

%#

%# The following commands define the symbols used to plot data points.

%#

/Pnt { stroke [] 0 setdash

gsave 1 setlinecap M 0 0 V stroke grestore } def

/Dia { stroke [] 0 setdash 2 copy vpt add M

hpt neg vpt neg V hpt vpt neg V

hpt vpt V hpt neg vpt V closepath stroke

Pnt } def

%#

%# ...and a bunch (dozens) more.

%#

%# The 'MF...' commands are used to handle the 'enhanced' syntax. If the

%# 'enhanced' mode is not invoked, these commands won't appear in the file.

%#

/MFshow {{dup dup 0 get findfont exch 1 get scalefont setfont

[ currentpoint ] exch dup 2 get 0 exch rmoveto dup dup 5 get exch 4 get

{show} {stringwidth pop 0 rmoveto}ifelse dup 3 get

{2 get neg 0 exch rmoveto pop} {pop aload pop moveto}ifelse} forall} bind def

/MFwidth {0 exch {dup 3 get{dup dup 0 get findfont exch 1 get scalefont setfont

5 get stringwidth pop add}

{pop} ifelse} forall} bind def

/MLshow { currentpoint stroke M

0 exch R MFshow } bind def

/MRshow { currentpoint stroke M

exch dup MFwidth neg 3 -1 roll R MFshow } def

/MCshow { currentpoint stroke M

exch dup MFwidth -2 div 3 -1 roll R MFshow } def

end

%#

%# The dictionary is now complete. We activate it, save what went before

%# (the 'gsave' command saves everything on a different stack) and begin.

%#

%%EndProlog

%%Page: 1 1

gnudict begin

gsave

%#

%# First we position the plot on the page and scale it.

%#

%# The 'translate' command moves the origin to the specified position.

%# [The PostScript default origin is near the lower left-hand corner.]

%# The 'scale' command changes the units used in the plot.

%# [The PostScript default unit is the point: 72 points equal one inch.]

%# The 'rotation' command rotates the coordinates clockwise through the

%# specified angle (degrees).

%# [The PostScript default orientation is profile.]

%#

%# This sample changes the orientation to landscape (the gnuplot default)

%# and the unit to tenths of a point. Note that if the first two commands

%# were interchanged, the translation would be only five points in each

%# direction, instead of fifty.

%#

%# If this had been generated in 'eps' mode, the "90 rotate" and "0 -5040

%# translate" commands would not appear and the units would be 0.050 instead

%# of 0.100.

%#

%# If you want to change the size or the position of the plot, this is where

%# to do it.

%#

50 50 translate

0.100 0.100 scale

90 rotate

0 -5040 translate

0 setgray

newpath

%#

%# Define the default font. The number is the height. As usual, fonts

%# used primarily for text have characters only about 70% the specified

%# height, because spacing between lines is built-in. The Symbol font and

%# others that are normally not used for text produce characters that are

%# more nearly the specified height. Thus if you want to intersperse, for

%# example, Greek and Roman letters (as you might when writing an equation),

%# you'll need to play with the heights of the two fonts in order for them

%# to look reasonably proportioned.

%#

%# The font defined here is used for the tick labels first, then the axis

%# labels and plot titles, and finally the key. If you want to change the

%# font, simply insert the modified line after you are done with the old one

%# (i.e. the last 'show' command to use it) but before the new one is needed.

%# Note that if you change the font height, you'll also want to change the

%# value of 'vshift' (it's near the top of the dictionary). I'll give an

%# example of this later on...

%#

(Helvetica) findfont 140 scalefont setfont

LTb

%#

%# Label and draw the ticks along the y-axis. I've given you three

%# y-tics in different formats...

%#

%# The tick length is 63 units.

%#

%# This first y-tic is in 'normal' mode:

%#

728 560 M

63 0 V

6325 0 R

-63 0 V

644 560 M

(-10) Rshow

%#

%# This y-tic is in 'enhanced' mode:

%#

728 1645 M

63 0 V

6325 0 R

-63 0 V

stroke

644 1645 M

[ [(Helvetica) 140.0 0.0 true true (-5)]

] -46.7 MRshow

%#

%# This one is in 'normal' mode, but is rotated:

%#

434 2730 M

63 0 V

6619 0 R

-63 0 V

-6759 0 R

currentpoint gsave translate 90 rotate 0 0 M

(0) Cshow

grestore

%#

%# Now the x-tics. Here's one:

%#

728 560 M

0 63 V

0 4277 R

0 -63 V

stroke

728 420 M

[ [(Helvetica) 140.0 0.0 true true (-10)]

] -46.7 MCshow

LTb

%#

%# Now draw the left and lower axes. Were this drawn by 'splot'

%# instead of 'plot', there might be some other axes drawn.

%#

728 560 M

6388 0 V

0 4340 V

-6388 0 V

728 560 L

stroke

%# Now come labels (both for the axes and those on "set label" commands)

%# and other titles. I just give you axis labels here, in 'enhanced' mode:

%#

140 2730 M

currentpoint gsave translate 90 rotate 0 0 moveto

[ [(Helvetica) 140.0 0.0 true true (ylabel)]

] -46.7 MCshow

grestore

3922 210 M

[ [(Helvetica) 140.0 0.0 true true (xlabel)]

] -46.7 MCshow

%#

%# We're finally ready to plot functions and/or data.

%#

%# The key is drawn just before the applicable data.

%#

%# Choose a line type and write the key.

%# In this sample, it is drawn with lines.

%#

1.000 UL

LT0

6465 4767 M (x) Rshow	%# the function label in the key

6549 4767 M	%# the sample line in the key

399 0 V

%#

%# Now the data (this is drawn with some combination of absolute and

%# relative lineto's and moveto's):

%#

728 560 M

2043 560 V

4926 911 L

2043 560 V

7116 4900 L

%#

%# ...and more.

%#

%# We don't need to eplicitly 'stroke' to draw the lines for each function

%# because the 'stroke' command is included in the line-type definition

%# commands. Thus switching line types automatically 'strokes' previous

%# lines. Clever program, that gnuplot...

%#

%# We can now repeat commands for the key and data for as many items

%# as were specified on the "plot" or "splot" command.

%#

%# Here's a second function, plotted with dots:

%#

LT1

6486 4486 M

(function 2) Rshow

6654 4486 Pnt

%#

%# The data:

%#

840 911 Pnt

2883 2030 Pnt

4926 2590 Pnt

6969 3710 Pnt

%#

%# ...and more.

%#

%# Since we're now done, we 'stroke' the last lines, close the dictionary

%# and restore the previous settings (those saved by the 'gsave' at the top).

%# [The 'gsave'/'grestore' pair is included so that if this file is embedded

%# in another PostScript file, this patch won't mess up the other parts of

%# the picture. It's considered good PostScript style to do this.]

%#

stroke

grestore

end

%#

%# And, finally, we send the page to the printer. [If we do embed this

%# file into another PostScript file, we'd remove this 'showpage'. Unless,

%# of course, this was appended to the other file, in which case we'd

%# remove the 'showpage' from the end of that file.]

%#

%#

showpage

%%Trailer

%%DocumentFonts: Helvetica

%%Pages: 1
